# Prompt and consistent improvement of coronary flow velocity reserve following successful recanalization of the coronary chronic total occlusion in patients with viable myocardium

**DOI:** 10.1186/s12947-020-00211-4

**Published:** 2020-07-21

**Authors:** Milan Dobric, Branko Beleslin, Milorad Tesic, Ana Djordjevic Dikic, Sinisa Stojkovic, Vojislav Giga, Miloje Tomasevic, Ivana Jovanovic, Olga Petrovic, Jelena Rakocevic, Nikola Boskovic, Dragana Sobic Saranovic, Goran Stankovic, Vladan Vukcevic, Dejan Orlic, Dragan Simic, Milan A. Nedeljkovic, Srdjan Aleksandric, Stefan Juricic, Miodrag Ostojic

**Affiliations:** 1 Cardiology Clinic, Clinical Center of Serbia, 26 Visegradska Street, Belgrade, 11000 Serbia; 2 Faculty of Medicine, University of Belgrade, 6 Dr Subotica Street, Belgrade, 11000 Serbia; 3 Department of Internal Medicine, Faculty of Medical Sciences, University of Kragujevac, 69 Svetozara Markovica Street, Kragujevac, 34000 Serbia; 4 Institute of Histology and Embryology, Faculty of Medicine, University of Belgrade, 6 Dr Subotica Street, Belgrade, 11000 Serbia

**Keywords:** Coronary chronic total occlusion, CTO, Recanalization, Coronary flow velocity reserve, CFVR

## Abstract

**Background:**

Coronary chronic total occlusion (CTO) is characterized by the presence of collateral blood vessels which can provide additional blood supply to CTO-artery dependent myocardium. Successful CTO recanalization is followed by significant decrease in collateral donor artery blood flow and collateral derecruitment, but data on coronary hemodynamic changes in relation to myocardial function are limited. We assessed changes in coronary flow velocity reserve (CFVR) by echocardiography in collateral donor and recanalized artery following successful opening of coronary CTO.

**Methods:**

Our study enrolled 31 patients (60 ± 9 years; 22 male) with CTO and viable myocardium by SPECT scheduled for percutaneous coronary intervention (PCI). Non-invasive CFVR was measured in collateral donor artery before PCI, 24 h and 6 months post-PCI, and 24 h and 6 months in recanalized artery following successful PCI of CTO.

**Results:**

Collateral donor artery showed significant increase in CFVR 24 h after CTO recanalization compared to pre-PCI values (2.30 ± 0.49 vs. 2.71 ± 0.45, *p* = 0.005), which remained unchanged after 6-months (2.68 ± 0.24). Baseline blood flow velocity of the collateral donor artery significantly decreased 24 h post-PCI compared to pre-PCI (0.28 ± 0.06 vs. 0.24 ± 0.04 m/s), and remained similar after 6 months, with no significant difference in maximum hyperemic blood flow velocity pre-PCI, 24 h and 6 months post-PCI. CFVR of the recanalized coronary artery 24 h post-PCI was 2.55 ± 0.35, and remained similar 6 months later (2.62 ± 0.26, p = NS).

**Conclusions:**

In patients with viable myocardium, prompt and significant CFVR increase in both recanalized and collateral donor artery, was observed within 24 h after successful recanalization of CTO artery, which maintained constant during the 6 months.

**Trial registration:**

ClinicalTrials.gov (Number NCT04060615).

## Introduction

Coronary chronic total occlusion (CTO) is defined as the complete occlusion of coronary artery with no epicardial blood flow in the occluded arterial segment, lasting more than three months [1–4]. It is characterized by the presence of more or less developed collateral blood vessels, and variable amount of viable myocardium in the perfusion territory of occluded artery [5, 6]. Although collateral blood vessels can adequately supply CTO artery-dependent myocardium in order to maintain its viability, or even contractility at rest, collaterals are often insufficient to supply blood during exercise [7].

After successful opening of CTO, coronary circulation undergoes early changes with reduction in collateral flow [8], reflected also by an increase in fractional flow reserve in collateral donor artery due to reduced perfusion territory [9]. Still, the data on changes in microvascular dysfunction reflected by coronary flow velocity reserve (CFVR), both in occluded and collateral donor artery are scarce [10]. In particular, Werner et al. [11] have shown that about 50% of patients after successful CTO opening still have microvascular dysfunction defined as invasive CFVR < 2.0, which improved over time in additional 50% of them. Improvement of CFVR over time was related to certain extent with improvement of wall motion score index, but not global ejection fraction. In general, the relation between microvascular dysfunction and myocardial function was not investigated in details, which is in contradiction with a need to define best patients who will gain most from the recanalization of CTO.

Noninvasive CFVR measured by 2D echocardiography represents versatile and highly reproducible noninvasive technique that may repeatedly interrogate coronary blood flow and microvascular dysfunction. Interestingly, this useful and user-friendly clinical noninvasive index was rarely used to assess hemodynamic and functional consequences following CTO opening [10]. In order to further define hemodynamic changes in coronary circulation, and relation to myocardial function, the aim of our study was to assess early and time-dependent changes in CFVR in occluded and non-occluded collateral donor artery after successful coronary CTO recanalization, in patients with viable myocardium in the territory of CTO as a prerequisite to perform PCI of CTO.

## Materials and methods

### Study population

This was a prospective, observational study conducted at the Cardiology Clinic, Clinical Center of Serbia, which enrolled 31 patients (22 male; mean age 60 ± 9 years), with CTO of coronary artery (right coronary artery, RCA - 22 patients, left anterior descending coronary artery, LAD - 8 patients, and left circumflex artery, LCx – 1 patient) scheduled for percutaneous recanalization. Inclusion criteria were: [1] age ≥ 18 years old, [2] one CTO present on native coronary artery, with CTO defined as a complete occlusion of coronary artery with no epicardial blood flow in the occluded arterial segment, lasting more than three months with, [3] viable myocardium in the territory of chronically occluded artery with a presence of either (a) typical anginal symptoms, or (b) inducible ischemia in the territory of chronically occluded artery, and [4] epicardial vessel at the occlusion location estimated ≥2.5 mm in diameter. Presence of viability in the myocardium supplied by the occluded artery was considered as the most objective and quantitative evidence of myocardium at risk for CTO recanalization. Exclusion criteria were: [1] acute coronary syndrome during previous 30 days, [2] contraindication to dual antiplatelet therapy for 12 months, [3] contraindication to drug-eluting stent (DES) implantation, [4] presence of more than one CTO on native coronary arteries, [5] presence of left ventricle wall aneurysm, [6] previous myocardial infarction in the territory of non-CTO coronary artery, [7] significant stenosis (more than 70% diameter stenosis) of non-CTO coronary artery requiring PCI [7]; previous coronary artery bypass graft surgery (CABG) or other cardiac surgical procedure, [8] left ventricle ejection fraction (LVEF) < 30%, [9] permanent atrial fibrillation, [10] pregnancy, [11] serum creatinine > 180 μmol/L (2 mg/dL), [12] patients unable/unwilling to cooperate and attend clinical visits during the follow-up period. All the procedures were in accordance with the “Declaration of Helsinki” and the ethical standards of the Ethical committee of Faculty of Medicine, University of Belgrade. Informed consent was obtained from each patient.

### Diagnostic evaluation before PCI

Before scheduled PCI for CTO, we have performed comprehensive functional evaluation to confirm a need for CTO opening. Thus, in all patients we have performed 2D transthoracic echocardiography, stress echocardiography for detection of myocardial ischemia, and single photon emission tomography myocardial perfusion imaging (SPECT MPI) for the detection of viable myocardium. In addition, for the purpose of the present study we have performed noninvasive transthoracic Doppler echocardiography to assess CFVR of the collateral donor artery (LAD or RCA) before PCI, as well as after PCI in both occluded and collateral donor arteries. In addition, to confirm net clinical benefit after CTO opening patients underwent quality of life evaluation using Seattle Angina Questionnaire (SAQ).

Initial 2D transthoracic echocardiography was used to evaluate regional left ventricular function. Wall motion score index (WMSI) was calculated using 17-segment model, with segmental kinetic grading score: 1 – normokinesia, 2 – hypokinesia, 3 – akinesia, and 4 – dyskinesia. WMSI was calculated by dividing the sum of the wall motion scores of each segment by total number of visualized segments [12]. Stress echocardiography exercise test was performed according to the submaximal Bruce treadmill protocol. Test was considered positive in the presence of exercise–induced wall motion abnormalities consistent with myocardial region supplied by occluded artery. Heart rate recovery was defined as a difference between maximum achieved heart rate during the test and heart rate after 1 min of recovery [13].

Noninvasive transthoracic Doppler echocardiography for the assessment of CFVR of the occluded and collateral donor artery was performed at rest and after the induction of hyperemia by intravenous infusion of adenosine (0.140 mg/kg/min). CFVR was calculated as a ratio of maximum hyperemic and maximum baseline blood flow velocities [14]. The intake of xanthine-containing foods or beverages was discontinued the day before the examination.

Gated SPECT MIBI was performed in all patients before PCI attempt using 466 ± 49 MBq (median 466 MBq) (range 407-555 MBq, 95% CI 450.1–483.5 MBq) of technetium-99 m-methoxyisobutylisonitrile (^99m^Tc-MIBI) intravenously, 10–15 min after giving 0.5 mg of sublingual nitroglycerine [15]. The SPECT acquisition was performed 45–60 min after the injection, using single head SPECT camera (Siemens ecam, Hoffman Estates, Illinois, USA) in 64 projections over a 180^0^ semicircular arc extending from 45^0^ right anterior oblique position to 45^0^ left posterior oblique position. Size and the extent of perfusion defect were calculated based on 17-segment model, according to the European Association of Nuclear Medicine, European Society of Cardiology guidelines for radionuclide imaging of cardiac function using 4D-MSPECT software [15, 16]. Total score of MIBI uptake, Summed Rest Score (SRS), was calculated. Perfusion defect was expressed according to the territory of three major coronary blood vessels: LAD, RCA, and LCx. Myocardium was considered viable if MIBI uptake was ≥55% of the normal perfusion, with preserved wall thickening [15, 17]. We also evaluated left ventricular ejection fraction using 4D-MSPECT automated algorithm for the determination of left ventricle surfaces from gated SPECT MPI [18].

SAQ is sensitive, valid and reproducible instrument used to assess the quality of life in patients with coronary artery disease [19]. This questionnaire is based on five different domains with scales reflecting patient’s physical limitation, angina stability, angina frequency, treatment satisfaction, and quality of life. Each scale is transformed into score (0–100), where higher score values indicate better results and better function. SAQ was assessed before PCI of CTO, and 6 months after the CTO recanalization.

### Percutaneous coronary intervention (PCI)

In all patients we utilized dual arterial access in order to optimally visualize the occluded artery and collateral blood vessels, and to plan the procedure. At the beginning of the procedure we applied nitroglycerine intracoronary (200 μg) and intravenous heparin (100 IU/kg). PCI for CTO was initially attempted using antegrade approach, which was immediately switched to retrograde in case of anterograde failure, using standard devices and approaches for these types of interventions [5, 11]. After successful passage of the coronary wire and balloon pre-dilatation, PCI with implantation of coronary drug eluting stents was performed. Successful procedure was defined as a restoration of TIMI 3 flow in previously occluded artery with residual stenosis < 30% after drug eluting stent implantation.

### Examinations after PCI

After 24 h following successful PCI, noninvasive transthoracic Doppler echocardiography with CFVR measurements for both LAD and RCA was performed. CFVR measurement in LAD artery was feasible in all patients (100%), whereas Doppler signal in RCA flow was of suboptimal quality in 2 patients, not allowing calculation of CFVR (feasibility 93%). Six months after the intervention, we have repeated non-invasive CFVR measurement for both LAD and RCA, and SAQ. All patients had regular clinical visits (at 1, 6, and 12 months), and follow-up for the appearance of major adverse cardiovascular events (MACE: sudden cardiac death, nonfatal myocardial infarction, and repeated revascularization).

### Statistical analysis

Sample size was calculated using the formula for paired sample Student’s t-test, based on the previously published studies and the study power of 0.8 to detect expected increase in CFVR in collateral donor artery after recanalization of CTO of 0.5 [10, 20, 21], with the statistical significance level of 0.05, and expected standard deviation for noninvasive Doppler CFVR in reference artery of 0.85 [22]. Calculated sample size was 23. With the current success rate of CTO recanalization at Cardiology Clinic, Clinical Center of Serbia of approximately 90%, as well as the expected 6-month follow-up drop-out of 15% of patients, optimal sample size was calculated to be 30 patients. Continuous variables are expressed as mean ± standard deviation, or median and interquartile range (IQR), where appropriate. Categorical variables are expressed as frequencies and percentages. Differences between the groups were tested using chi-square test, paired sample Student’s t-test, independent sample t-test, and repeated measures ANOVA where appropriate. Association between variables was tested using Pearson’s correlation. Statistical analyses were performed using IBM SPSS Statistics version 25 for Windows (IBM Corporation, Armonk, NY, USA).

## Results

Patients’ baseline and angiographic characteristics are summarized in Table 1. Fourteen (45.2%) patients had previous myocardial infarction, while stress echocardiography test was positive for inducible myocardial ischemia in 21 (67.7%) patients, and negative in 10 (32.3%) patients.

**Table 1:** Patients’ baseline and angiographic characteristics (*N* = 31 patients, whole study group)

Baseline characteristics	
Age, years (mean ± SD)	60 ± 9
Male, n (%)	22 (71)
BMI, kg/m^2^ (mean ± SD)	27.6 ± 3.8
Hypertension, n (%)	27 (87.1)
Hyperlipidemia, n (%)	29 (93.5)
Diabetes mellitus	9 (29.0)
Smoking	19 (61.3)
Family history of cardiovascular disease, n (%)	16 (51.6)
Previous myocardial infarction, n (%)	14 (45.2)
Previous PCI, n (%)	10 (32.3)
Stress echocardiography test	
• Positive, n (%)	21 (67.7)
• Negative, n (%)	10 (32.3)
Acetylsalicylic acid, n (%)	31 (100)
P2Y12 inhibitor, n (%)	31 (100)
ACE inhibitors, n (%)	20 (64.5)
ARBs, n (%)	1 (3.2)
Beta blockers, n (%)	24 (77.4)
Trimetazidine, n (%)	13 (41.9)
Long-acting nitrates, n (%)	9 (29.0)
CCBs, n (%)	9 (29.0)
Statins, n (%)	25 (80.6)
Diuretics, n (%)	7 (22.6)

*SD* standard deviation*, BMI* body mass index*, PCI* percutaneous coronary intervention*, ACE* angiotensin converting enzyme*, ARB* angiotensin II receptor blockers*, CCB* calcium channel blockers

The majority of patients had chronically occluded RCA (22 patients, 71.0%), 8 (25.8%) patients had CTO of the LAD, while only one patient (3.2%) had CTO of the LCx. Collateral-donor artery was LAD in 23 (74.2%) patients, and RCA in 8 (25.8%) patients. Single-vessel disease was present in the majority of patients (23 patients, 74.2%). Six (19.4%) patients had two-vessel disease, while 2 (6.5%) patients had 3-vessel disease (lesions in non-occluded coronary arteries were mild to moderate and not considered significant to perform PCI).

CTO recanalization procedure was successful in 28 (90.3%) patients, and this was final study population involved in coronary physiology assessment defined by study protocol. Median number of implanted DES per patient was 2 (IQR 1–3). Median length of implanted stents was 51 mm (IQR 31.75–74.5 mm), and median stent diameter was 3.0 mm (IQR 3.0–3.5 mm).

### SPECT and stress echocardiography data

Patients’ SPECT and echocardiography data are shown in Table 2. SPECT MPI showed the presence of viable myocardium in all patients. Mean LVEF measured by gated SPECT MPI was 57 ± 10%. Stress echocardiography test was positive in 18 (64.3%) patients. Median number of visualized ischemic segments during the test was 2. Chest pain was recorded during the test in 7 (25%) patients.

**Table 2:** Patients’ SPECT and echocardiography data (*n* = 28 patients with successful CTO recanalization)

SPECT MPI	
LVEF (mean ± SD)	57 ± 10%
SRS (median, IQR)	5 (1–12)
Perfusion abnormality (median, IQR)	8.5 (1.5–19.5)
**Stress echocardiography**	
Test result	
• Positive (n, %)	18 (64.3%)
• Negative	10 (35.7%)
Ischemic myocardial segments (median, IQR)	2 (0–3)
Chest pain during the test (n, %)	7 (25%)
WMSI at rest	1.139 ± 0.166
WMSI at peak stress	1.301 ± 0.275
LVEF at rest (%)	55.9 ± 9.7
LVEF at peak stress (%)	53.1 ± 11.1
Heart rate recovery (bpm, mean ± SD)	36.8 ± 10.2
Duke score (median, IQR)	2.0 (− 1.5 to 3.5)

*LVEF* left ventricle ejection fraction*, SD* standard deviation*, SRS* summed rest score*, IQR* interquartile range*, WMSI* wall motion score index*, LVEF* left ventricle ejection fraction*, bpm* beats per minute

### CFVR of the collateral donor artery

Pre-procedural noninvasive transthoracic Doppler CFVR of the collateral donor artery was 2.30 ± 0.49, which increased to 2.71 ± 0.45 24 h after the recanalization of CTO (Table 3). There was no difference in pre-PCI CFVR values between patients where LAD or RCA was the collateral donor artery (2.39 ± 0.53 vs. 2.08 ± 0.28, respectively, *p* = 0.780), with no difference in CFVR after 24 h (2.73 ± 0.48 vs. 2.64 ± 0.35, respectively, p = 0.780). After 6 months, CFVR of the collateral donor artery was 2.68 ± 0.24, with no difference between LAD and RCA (2.70 ± 0.25 vs. 2.64 ± 0.23, respectively, *p* = 0.621).

**Table 3:** Temporal changes in CFVR, maximum baseline blood flow, and maximum hyperemic blood flow in collateral donor artery and recanalized coronary artery (n = 28 patients with successful CTO recanalization)

	Collateral donor artery	Recanalized artery
CFVR pre-PCI	2.30 ± 0.49	/
• Maximum baseline blood flow velocity (m/s)	0.28 ± 0.06	/
• Maximum hyperemic blood flow velocity (m/s)	0.64 ± 0.1	/
CFVR 24 h after PCI	2.71 ± 0.45	2.55 ± 0.35
• Maximum baseline blood flow velocity (m/s)	0.24 ± 0.04	0.28 ± 0.07
• Maximum hyperemic blood flow velocity (m/s)	0.64 ± 0.16	0.71 ± 0.18
CFVR 6 months after PCI	2.68 ± 0.24	2.62 ± 0.26
• Maximum baseline blood flow velocity (m/s)	0.26 ± 0.05	0.26 ± 0.03
• Maximum hyperemic blood flow velocity (m/s)	0.70 ± 0.16	0.69 ± 0.09

*CFVR* coronary flow velocity reserve

**Fig. 1:**
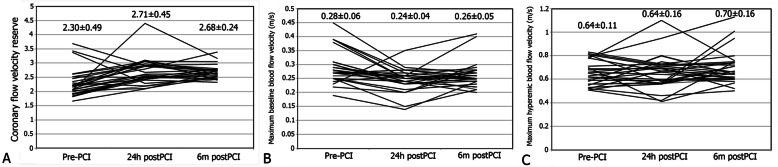
Temporal change in CFVR (**a**), maximum baseline blood flow velocity (**b**), and maximum hyperemic blood flow velocity of the donor artery (**c**) before PCI, 24 h after, and 6 months after PCI, *values represent mean ± standard deviation

There was a significant difference in measured CFVR levels pre-PCI, 24 h after, and 6 months after PCI (F = 392.181, *p* < 0.001) (Table 3, Fig. 1a). Post hoc comparisons revealed significant increase in collateral donor artery CFVR 24 h after CTO recanalization as compared to pre-PCI values (*p* = 0.005), remaining at the same level during 6-months follow up (*p* = 1.000 for the 6-months vs. 24-h comparison).

We found significant difference in Doppler derived maximum baseline blood flow velocity of the collateral donor artery measured before PCI, 24 h after, and 6 months after PCI (Table 3, Fig. 1b). Post hoc comparisons showed that there was significant decrease of Doppler derived maximum baseline blood flow velocity of the collateral donor artery measured 24 h after intervention, as compared to pre-PCI values (*p* = 0.011), and that this velocity remained similar during the 6-months follow-up (*p* = 0.182 for the 6-months vs. 24-h comparison). There was no difference in maximum hyperemic blood flow velocity of the collateral donor artery measured before PCI, 24 h after, and 6 months after PCI (F = 2.007, *p* = 0.148) (Table 3, Fig. 1c).

As opposed to most, three patients demonstrated marked decrease in CFVR of the collateral donor artery 24 h after the procedure compared to prePCI values (from baseline 3.38 to 2.28, from 3.68 to 2.92, and from 3.43 to 2.68; Fig. 1a). In two patients observed decline was largely driven by the decrease in maximum hyperemic blood flow velocity (from 0.81 to 0.58 m/s, and from 0.70 to 0.41 m/s). In third patient both baseline and hyperemic velocities increased (from 0.23 to 0.35 m/s, and from 0.79 to 0.84 m/s, respectively). Analyzing clinical, angiographic, and procedural characteristics, we could not find the possible explanation for the observed CFVR decrease in collateral donor artery.

**Fig. 2:**
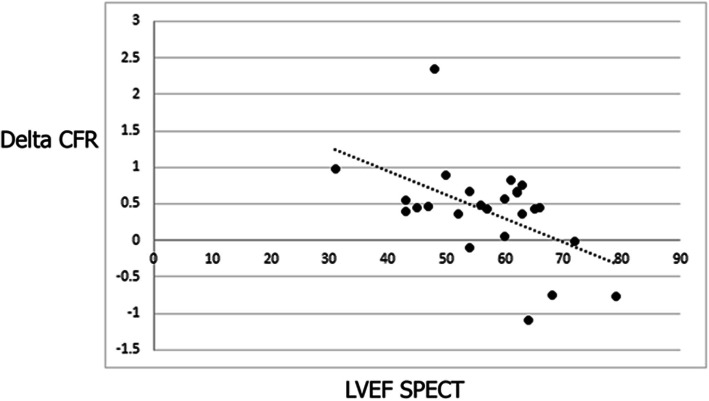
Inverse correlation between LVEF measured by gated SPECT MPI (x-axis) and change (delta) in CFVR of the collateral donor artery 24 h post-PCI compared to pre-PCI values (y-axis)

Change (delta) in CFVR of the collateral donor artery 24 h post-PCI compared to pre-PCI values showed inverse correlation with LVEF measured by gated SPECT MPI (r = − 0.523, *p* = 0.007, Fig. 2), but showed no correlation with WMSI on transthoracic echocardiography (*p* = 0.405), nor correlation with patients’ initial stress echocardiography test result (*p* = 0.120), gender (*p* = 0.782), smoking status (*p* = 0.487), presence of dyslipidemia (*p* = 0.893), diabetes (*p* = 0.839), or previous myocardial infarction (*p* = 0.858). We further investigated the relation of stress-test induced changes in left ventricular function to hemodynamic changes induced by CTO recanalization. We correlated changes (delta) in LVEF and WMSI during stress echo test (baseline minus peak stress values) to non-invasive hemodynamic parameters in collateral donor and CTO arteries, and found that only maximum hyperemic blood flow achieved in CTO artery 24 h after recanalization was related to change (decrease) in LVEF (ρ = 0.413, *p* = 0.029, Table 4).

**Table 4:** Correlation of changes in LVEF and WMSI during stress echo test and flow parameters in collateral donor and CTO arteries (*n* = 28 patients with successful CTO recanalization)

	Delta LVEF (baseline minus peak stress)	Delta WMSI (baseline minus peak stress)
**Before PCI - collateral donor artery**		
*CFVR*	ρ = 0.214, *p* = 0.275	ρ = 0.080, *p* = 0.687
*Maximum baseline blood flow*	ρ = −0.162, *p* = 0.409	ρ = −0.251, *p* = 0.197
*Maximum hyperemic blood flow*	ρ = 0.232, *p* = 0.236	ρ = − 0.098, *p* = 0.619
**24hafter PCI - collateral donor artery**		
*CFVR*	ρ = −0.015, *p* = 0.940	ρ = −0.181, *p* = 0.358
*Maximum baseline blood flow*	ρ = 0.169, *p* = 0.390	ρ = 0.082, *p* = 0.680
*Maximum hyperemic blood flow*	ρ = −0.029, *p* = 0.884	ρ = 0.204, *p* = 0.298
*Delta CFVR donor artery (24 h* vs. *baseline)*	ρ = −0.214, *p* = 0.274	ρ = 0.229, *p* = 0.241
**24 h after PCI - CTO artery**		
*CFVR*	ρ = 0.318, *p* = 0.099	ρ = −0.028, *p* = 0.887
*Maximum baseline blood flow*	ρ = 0.302, *p* = 0.119	ρ = −0.376, *p* = 0.059
*Maximum hyperemic blood flow*	ρ = 0.413, p = 0.029 *	ρ = −0.155, *p* = 0.431

*PCI* percutaneous coronary intervention*, CFVR* coronary flow velocity reserve; * p < 0.05

**Fig. 3:**
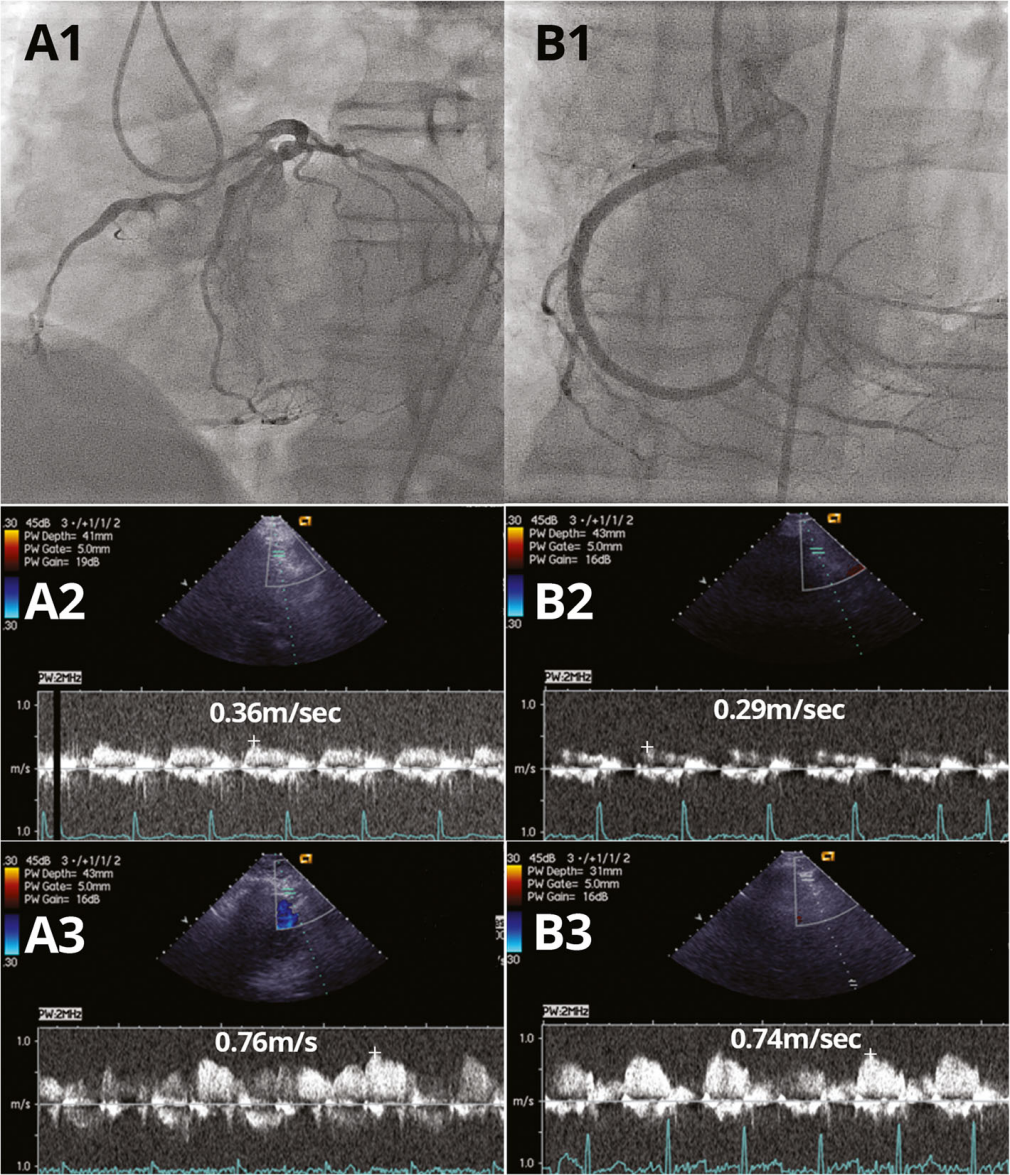
**a** Before CTO RCA recanalization: CTO of RCA with collaterals from LAD (A1); peak baseline (A2) and hyperemic (A3) diastolic flow velocities in collateral donor LAD artery - CFVR LAD (0.76/0.36) - 2.11. **b** After CTO RCA recanalization: Recanalized RCA (B1); peak baseline (B2) and hyperemic (B3) diastolic flow velocities in LAD artery - CFVR LAD (0.74/0.29) - 2.55

Example of CFVR pattern in collateral donor artery before and after CTO recanalization along with the corresponding angiographic finding is presented on Fig. 3.

### CFVR of the recanalized coronary artery

CFVR of the recanalized coronary artery 24 h after PCI was 2.55 ± 0.35, with no difference between LAD and RCA (2.49 ± 0.35 vs. 2.69 ± 0.32, respectively, *p* = 0.188). After 6 months, CFVR of the recanalized artery was 2.62 ± 0.26, which was almost the same as 24 h after successful PCI (Table 3, *p* = 0.279). Six months after PCI, patients in whom RCA was recanalized had higher CFVR than patients in whom LAD was opened (2.84 ± 0.31 vs. 2.54 ± 0.18, *p* = 0.003).

CFVR values of the recanalized artery 24 h after the intervention showed no correlation with LVEF measured on SPECT (*p* = 0.332), WMSI on transthoracic echocardiography (*p* = 0.343), nor ischemia during stress echocardiography test (*p* = 0.836). Interestingly, CFVR of the CTO artery measured 24 h after PCI was not related to other patients’ characteristics (gender, smoking, hyperlipidemia, diabetes, and previous myocardial infarction).

### SAQ measurements and clinical follow-up

Patients reported improvements in four out of five SAQ domains during the 6 month follow up, as compared to pre-PCI values, while the fifth domain (anginal stability scale) showed borderline significance (Table 5).

**Table 5:** Values of SAQ domains 6 months after CTO recanalization vs. pre-PCI values (*n* = 28 patients with successful CTO recanalization)

SAQ	Baseline	6 months follow-up	p
Physical limitation	68.31 (51.86–79.87)	85.49 (53.39–90.97)	0.019
Angina stability	50.00 (0–50.00)	50.00 (31.25–50.00)	0.051
Angina frequency	65 (30–95)	90 (50–100)	0.008
Treatment satisfaction	81.25 (65.63–100.00)	100.00 (81.25–100.00)	0.009
Quality of life	54.16 (41.67–72.92)	87.50 (66.68–100.00)	0.001

*SAQ* Seattle Angina Questionnaire

Neither change in CFVR nor change in maximum baseline blood flow velocity of the collateral donor artery (6 months vs. baseline) or recanalized CTO artery was correlated to numeric change (delta) in five SAQ domains.

During 1, 6, and 12-month clinical follow-up, none of the study patients experienced major adverse cardiovascular events.

## Discussion

To the best of our knowledge, this is the first study to evaluate noninvasive CFVR by 2D echocardiography in both donor and recanalized artery, demonstrating early and rapid restoration of blood flow in recanalized artery with compensatory reduction in resting blood flow in collateral donor artery. In particular, our study demonstrated that in patients with viable myocardium undergoing PCI of CTO, successful recanalization of occluded artery induced prompt restoration of CFVR in recanalized artery and significant increase in CFVR of the collateral donor artery within 24 h, which maintained consistent and constant after 6 months follow-up. Increase in CFVR in collateral donor artery was largely driven by the significant reduction in the maximum baseline blood flow velocity within 24 h after CTO recanalization compared to pre-PCI values, while the maximum hyperemic blood flow velocity measured pre-PCI, 24 h after PCI and 6 months after PCI remained similar. It is of particular interest to note that no interaction was observed between CFVR improvement and previous myocardial infarction or diabetes mellitus that might induce microvascular dysfunction and blunt the effect of CTO recanalization, outlining the importance of myocardial viability for CTO recanalization.

If the perfusion territory is largely supplied by the collateral flow from the donor artery, CTO recanalization and restoration of anterograde flow results in the prompt reduction or even cessation of collateral flow. This could lead to the reduction of blood flow in the donor artery as well, which could be reflected as a change in hemodynamic parameters of the donor artery (e.g. fractional flow reserve, FFR). Previous study showed that the collateral flow supplies approximately 50% of the anterograde flow restored after the CTO recanalization [23]. Several trials have observed the increase in FFR of the donor artery after the successful recanalization of chronically occluded artery, which in some cases altered the FFR values so that initial FFR values of < 0.8 increased and became functionally insignificant, requiring no revascularization [8, 24–29].

Coronary flow reserve evaluation either by invasive or non-invasive means, reflects both epicardial and microvascular compartment of coronary circulation, and have excellent negative predictive potential, meaning that when CFVR is > 2, or particularly > 2.5, both epicardial and microvascular beds are functioning normally [30, 31]. However, invasive evaluation of CFVR is available and performed only in very sophisticated centers, and measurement per se might be quite demanding and less feasible in certain patients [32, 33]. On the other hand, non-invasive measurement of coronary blood flow velocity with CFVR assessed by transthoracic Doppler echocardiography is feasible and quite reproducible [34]. Nevertheless, evaluation of CFVR is generally more feasible in the LAD (above 95%) [35–37] as compared to the RCA, for which feasibility goes from 85% [35] to 90% [37]. Interestingly, there are just a few data on CFVR interrogation by non-invasive 2D echocardiography in CTO patients. Pizzuto et al. [38] explored with just a single CFVR measurement coronary flow of the collateral donor artery within 24-48 h of the diagnostic coronary angiography, and found that CFVR values showed positive correlation with the degree of angiographically visible collaterals and the number of diseased vessels. However, CFVR values were not measured after CTO recanalization. Baykan et al. [10] evaluated only changes in CFVR of the donor artery before and after CTO recanalization, and observed significant increase in CFVR of the donor artery 3 months after the CTO recanalization compared to pre-PCI and CFVR values measured 24 h post-PCI. In relation to our study, significantly lower values of CFVR were observed (almost in all patients under 2.0), which remained the same 24 h after PCI, but improved after 3 months. Our study showed significant increase in CFVR of the collateral donor artery as early as 24 h after CTO recanalization, which remained at the same level after 6 months compared to pre-PCI values. Different values and time changes in CFVR may be explained by the different population of patients including risk factors, previous myocardial infarction and other coronary stenosis in the donor artery.

Percutaneous treatment of coronary CTO represents one of the most challenging coronary lesions for operators, with several treatment algorithms among different CTO clubs [39], with the overall success rate in dedicated and expert centers of > 90%. Recently, international collaboration resulted in a global consensus document emphasizing several principles for good clinical practice of CTO recanalization [40], with ischemic symptoms improvement as the primary indication for PCI of CTO. Therefore, careful selection of patients that could gain the most benefit from these complex interventions should rely on documentation of anginal symptoms, myocardial ischemia and/or viability in the CTO territory [11]. Here, we have demonstrated that in patients with objective evidence of viable myocardium by SPECT, even with previous myocardial infarction or without clear stress echocardiography evidence of myocardial ischemia, there is a prompt restoration of microvascular function as reflected by early improvement in CFVR. Recanalization of CTO translates into slight improvement of regional and global ejection fraction [41, 42], but in case of viable myocardium there is a high probability to achieve better functional outcome [41], here confirmed by a correlation between changes in CFVR and SPECT assessed myocardial function.

Successful CTO revascularization was proved to be associated with improved quality of life [43, 44], which was confirmed in our study. Our patients reported significant improvement of quality of life measured by SAQ 6 months after the CTO recanalization. As there was no relation between improvement of CFVR or other parameters and SAQ changes, we may only hypothesize that quality of life perception is more complex than mere improvement in coronary physiology or function.

CTO recanalization is demanding procedure that is known for large radiation exposure to the patient and the operator. Usually, for the purpose of detecting viability of the myocardium in the territory of occluded artery, additional cardiovascular imaging techniques employing ionizing radiation (such as SPECT) are utilized. Taking these radiation exposures together, cumulative dose to which patient is exposed could be significant. In our study population, estimates of radiation exposure during SPECT were calculated from the 2015 International Commission on Radiological Protection Compendium for Tc-99 m with all doses then converted to an effective dose in mSv [45, 46]. Based on this information, we calculated patient radiation exposure of 0.009 mSv/MBq for Tc-99 m MIBI. For our group of patients mean radiation exposure was 4.1 ± 0.4 mSv (95% CI 4.0–4.3 mSv; range 3.66–4.99 mSv, median 3.99 mSv). In comparison to radiation exposure during chest X-ray which is about 0.02 mSv, this dose is equivalent to approximately 200 chest X-rays [47]. Average radiation dose (total air-kerma) during PCI in our population was 1344 mGy (IQR 967–2004 mGy).

Several study limitations are relevant to this study. First, we investigated highly selected group of patients with coronary CTO, viable myocardium in the territory of CTO, first attempt of CTO, and no-significant disease in the other coronary arteries, which may not adequately represent real-life clinical practice and all CTO lesions submitted for recanalization. Second, this was a single-center based study and it included relatively small number of participants. And finally, our study did not include control group of patients with CTOs treated conservatively. Finally, although non-invasive 2D echo CFVR is not utilized worldwide, our center has a large experience of CFVR measurements in different patients’ subsets with high feasibility and reproducibility [14, 20, 48].

## Conclusions

In patients with viable myocardium undergoing PCI of CTO, recanalization of CTO results in prompt and consistent restoration of coronary flow in recanalized coronary artery with rapid (within 24 h of PCI) improvement in coronary flow reserve of collateral donor artery, due to the reduction in maximum baseline blood flow velocity in donor artery and collateral derecruitment. Our results outline the importance of viability assessment in patients scheduled for PCI of CTO in order to gain most of recanalization of CTO.

## Data Availability

The datasets used and/or analyzed during the current study are available from the corresponding author on reasonable request.

## Abbreviations

CABG: Coronary artery bypass graft surgery
CFVR: Coronary flow velocity reserve
CTO: Chronic total occlusion
FFR: Fractional flow reserve
LAD: Left anterior descending coronary artery
LCx: Left circumflex artery
LVEF: Left ventricle ejection fraction
PCI: Percutaneous coronary intervention
RCA: Right coronary artery
SAQ: Seattle Angina Questionnaire
SPECT MPI: Single photon emission tomography myocardial perfusion imaging
WMSI: Wall motion score index
